# Seroprevalence of *Toxoplasma gondii* in commensal rodents sampled across Senegal, West Africa

**DOI:** 10.1051/parasite/2018036

**Published:** 2018-07-16

**Authors:** Carine Brouat, Christophe Amidi Diagne, Khadija Ismaïl, Abdelkrim Aroussi, Ambroise Dalecky, Khalilou Bâ, Mamadou Kane, Youssoupha Niang, Mamoudou Diallo, Aliou Sow, Lokman Galal, Sylvain Piry, Marie-Laure Dardé, Aurélien Mercier

**Affiliations:** 1 CBGP, IRD, CIRAD, INRA, Montpellier SupAgro, Univ. Montpellier 755 avenue du campus Agropolis 34988 Montferrier-sur-Lez cedex France; 2 BIOPASS, CBGP-IRD, ISRA, UCAD Campus de Bel-Air BP 1386 Dakar CP 18524 Senegal; 3 UMR-S 1094, Neuroépidémiologie Tropicale, INSERM, Univ. Limoges 2 rue du Dr Marcland 87025 Limoges France; 4 LPED, IRD, Aix Marseille Univ., Centre St Charles case 10, 3 place Victor Hugo CS 80249 13331 Marseille cedex 03 France

**Keywords:** Africa, rodents, Senegal, seroprevalence, *Toxoplasma gondii*

## Abstract

Risks related to *Toxoplasma gondii* infection in humans remain poorly known in Senegal. Although rodent surveys could help to assess the circulation of *T. gondii*, they have seldom been set up in sub-Saharan Africa. The aim of this study was to examine *Toxoplasma* seroprevalence in rodents from villages and towns across Senegal. Rodents were sampled in 40 localities using a standardised trapping protocol. Detection of *T. gondii* antibodies was performed on 1205 rodents, using a modified agglutination test (MAT) technique. Seroprevalence data were analysed depending on geography, the local rodent community, and individual characteristics of the rodent hosts. We found 44 seropositive rodents from four different species (*Mastomys erythroleucus, Mastomys natalensis*, *Mus musculus domesticus*, *Rattus rattus*). *Toxoplasma* seroprevalence was low, averaging 4% in the localities. Higher *Toxoplasma* seroprevalence (up to 24%) was found in northern Senegal, a region known to be the heart of pastoral herding in the country.

## Abstract (fr)

Les risques liés à l’infection par *Toxoplasma gondii* chez l’homme restent mal connus au Sénégal. Bien que les rongeurs soient considérés comme des marqueurs pertinents de la circulation de *T. gondii*, peu d’études ont ciblé ces hôtes en Afrique sub-saharienne. Le but de cette étude était d’étudier la séroprévalence de *Toxoplasma* chez les rongeurs des villes et des villages du Sénégal. Les rongeurs ont été échantillonnés dans 40 localités avec un protocole de piégeage standardisé. La détection des anticorps contre *T. gondii* a été faite sur 1205 rongeurs avec un test d’agglutination modifié. Les données de séroprévalence ont été analysées en fonction de la géographie, de la communauté locale de rongeurs et de caractéristiques individuelles du rongeur. Nous avons trouvé 44 individus séropositifs, de quatre espèces différentes (*Mastomys erythroleucus, Mastomys natalensis*, *Mus musculus domesticus*, *Rattus rattus*). La séroprévalence moyenne est faible, de l’ordre de 4 % au sein des localités. Les plus fortes séroprévalences (jusqu’à 24 %) ont été observées dans le Nord du Sénégal, une région connue pour être le cœur de l’élevage pastoral dans le pays.

## Introduction

*Toxoplasma gondii* [25] is an intracellular pathogen with a worldwide distribution [17]. In humans, infections range in severity from asymptomatic to lethal, and are particularly dangerous to the unborn child during pregnancy and to immunosuppressed patients. Transmission to humans may occur either congenitally, or by ingestion of undercooked meat containing tissue cysts, or of food and water contaminated with oocysts shed into the environment in the faeces of felids. Although felids are the only known definitive host of *T. gondii,* all homoeothermic animals may act as intermediate hosts [33]. Rodents constitute important prey for domestic felids, and are among the few wild mammals to persist in villages and towns. For this reason, they are often considered relevant markers to assess the circulation of *T. gondii* in commensal habitats [23, 30].

In sub-Saharan Africa, human seroprevalence is highly variable, with reported values ranging from 4% to 83% [20]. In Senegal, *T. gondii* infection in humans has been found to vary between 4% and 40% [19, 24]. However, most studies concerned the city of Dakar and the distribution of risks related to the disease across the country remains largely unknown. Moreover, the seroprevalence of *Toxoplasma* in animal populations has rarely been characterised in Senegal [8, 12].

In this study, we provide serological data for *T. gondii* obtained from 1205 rodents sampled in villages and towns in Senegal. Seroprevalence data were analysed regarding the geographical location of the sampling locality, its rodent community, and the individual characteristics of the rodent hosts.

## Material and methods

### Sampling

**Fig. 1.**
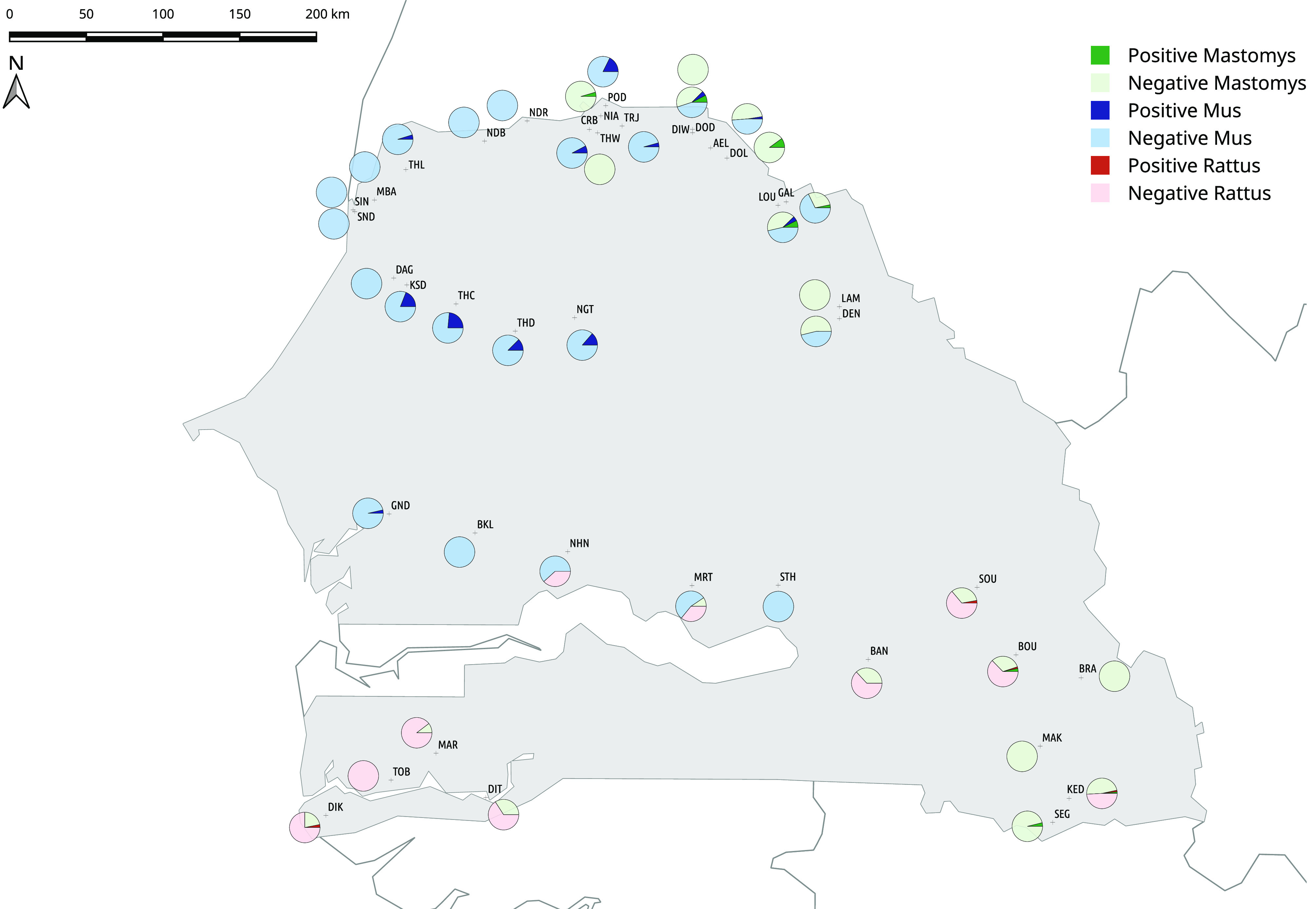
Seroprevalence of *Toxoplasma gondii* in rodent communities in villages and towns in Senegal. See Table 1 for locality codes. Light and dark colours indicated the percentage of negative (−) and positive (+) rodents of each dominant species in the community (*Mus musculus*, *Rattus rattus* or *Mastomys* spp.), respectively.

Fieldwork was conducted in 40 localities in Senegal between 2011 and 2014 (Fig. 1, Table 1). Trapping within private properties was performed with prior agreement from local authorities. All animal-related procedures were carried out under our laboratory authorisation for experiments on wild animals (No. D 34-169-1), and followed the official guidelines of the American Society of Mammalogists [31]. The detailed description of the standardised trapping protocol used here was provided in [7]. Rodents were captured alive and euthanised using cervical dislocation, weighed to the nearest 0.5 g, sexed and dissected. Intra-cardiac blood was sampled immediately after death and spotted onto Whatman No. 3 papers that were air-dried and then stored in a plastic bag at room temperature (RT) in the field and then at 4 °C in the laboratory.

**Table 1.** Rodent sampling in Senegal, number of seropositive individuals for *Toxoplasma gondii*, and mean seroprevalence per locality and rodent species.

Locality	Code	Long.	Lat.	*N* a	*N*_sero_ b	SP^c^ (%)	S^d^	T_S_ e	Rodent species^f^					
									*A. niloticus*	*M. erythroleucus*	*M. natalensis*	*P. daltoni*	*M. m. domesticus*	*R. rattus*
Aere Lao	AEL	−14.32	16.40	73	41	2.4	0.58	0.36		22\20\0			51\21\1	
Badi Nieriko	BAN	−13.38	13.38	89	38	0	0.72	0.41		14\14\0		01\00\–		74\24\0
Boutougoufara	BOU	−12.49	13.40	67	65	4.6	0.46	0.28	01\01\0	21\21\2		05\05\0		40\38\1
Bransan	BRA	−12.10	13.26	41	32	0	0.67	0.33	01\01\0	06\04\0	33\26\0	01\01\0		
Croisement Boube	CRB	−15.06	16.51	49	13	7.7	0.85	0.11	01\00\–	03\00\–			45\13\1	
Dagathie	DAG	−16.25	15.63	30	20	0	1.00	0.29					30\20\0	
Dendoudi	DEN	−13.54	15.39	49	39	0	0.47	0.20	03\00\–	28\21\0			18\18\0	
Diakene-Wolof	DIK	−16.64	12.46	39	33	3.0	0.63	0.36		08\07\0		01\01\0		30\25\1
Diattacounda	DIT	−15.68	12.57	52	45	0	0.55	0.42		16\15\0		01\01\0		35\29\0
Diomandou Walo	DIW	−14.43	16.51	15	14	0	0.88	0.08	01\00\–	14\14\0				
Dodel	DOD	−14.43	16.49	57	40	12.5	0.51	0.40		33\20\3			24\20\2	
Doumnga Lao	DOL	−14.22	16.34	46	20	10.0	0.96	0.49	01\00\–	45\20\2				
Galoya	GAL	−13.86	16.08	94	31	3.2	0.66	0.32	05\00\–	14\10\1			75\21\0	
Gandiaye	GND	−16.27	14.24	29	28	3.6	0.93	0.26					28\28\1	
Kedougou	KED	−12.18	12.55	65	59	3.4	0.50	0.52			30\29\1			35\30\1
Keur Seyni Dieng	KSD	−16.17	15.59	23	23	17.4	0.84	0.22	02\02\0				21\21\4	
Lambago	LAM	−13.54	15.46	24	20	0	0.92	0.07	01\00\–	23\20\0				
Lougue	LOU	−13.91	16.06	56	41	12.2	0.47	0.41	02\00\–	26\20\3			28\21\2	
Mako	MAK	−12.35	12.86	52	26	0	0.86	0.46			48\26\0	04\00\–		
Marsassoum	MAR	−15.98	12.83	34	30	0	0.74	0.41		03\03\0		02\01\0		29\26\0
Mbakhana	MBA	−16.37	16.09	30	23	0	1.00	0.28					30\23\0	
Mbirkilane	BKL	−15.75	14.13	32	32	0	1.00	0.42					32\32\0	
Mereto	MRT	−14.44	13.82	68	62	0	0.33	0.37	09\09\0	06\05\0			30\29\0	23\19\0
Ndiareme	NDR	−15.44	16.56	26	24	0	0.86	0.33		02\00\–			24\24\0	
Ndombo	NDB	−15.70	16.44	24	20	0	0.92	0.16	01\00\–				23\20\0	
Nguith	NGT	−15.15	15.40	22	22	13.6	1.00	0.30					22\22\3	
Niahene	NHN	−15.19	14.02	36	35	0	0.50	0.27	01\01\0				22\21\0	13\13\0
Niandane	NIA	−14.99	16.59	45	44	4.5	1.00	0.49		45\44\2				
Podor	POD	−14.96	16.65	18	17	17.6	0.90	0.21					17\17\3	
Segou	SEG	−12.28	12.41	31	31	3.2	0.73	0.27			26\26\1	05\05\0		
Sinthiou Maleme	STH	−13.92	13.82	30	19	0	1.00	0.29					30\19\0	
Soutouta	SOU	−12.72	13.80	49	46	2.2	0.38	0.26	10\09\0	12\12\0		01\01\0		26\24\1
Saint-Louis Ile Nord	SIN	−16.50	16.03	29	25	0	0.87	0.22					27\25\0	
Saint-Louis Sor	SND	−16.49	16.02	26	24	0	1.00	0.41					26\24\0	
Taredji	TRJ	−14.86	16.53	26	23	4.3	1.00	0.37					26\23\1	
Thiamene Cayor	THC	−15.87	15.48	17	17	23.5	1.00	0.16					17\17\4	
Thiamene Djolof	THD	−15.51	15.32	17	16	12.5	1.00	0.15					17\16\2	
Thiewle	THW	−15.01	16.49	34	22	0	1.00	0.34		34\22\0				
Thilène	THL	−16.18	16.27	26	21	4.8	1.00	0.31					26\21\1	
Tobor	TOB	−16.25	12.67	34	24	0	0.74	0.43		01\00\–		04\03\0		29\21\0
Total				16041205					39\23\0	376\292\13	137\107\2	25\18\0	689\516\25	334\249\4
Mean seroprevalence (%)						4.2			0.0	4.5	1.9	0	4.8	1.6

a Number of sampled rodents;

b Number of rodents considered for serological analyses;

c Seroprevalence of *T. gondii*;

d Simpson’s diversity index;

e Trap success, calculated as the ratio between the number of rodents sampled and the number of traps set in the houses, minus non-active traps (e.g., those found closed and empty, or those having captured small mammals other than rodents);

f Number of individuals sampled/Number of individuals serotyped/Number of seropositive individuals.

Rodents were identified to the species level using morphologic tools [14]. The rodent community was characterised at each locality using trap success (e.g., the ratio between the number of captured rodents and active traps set), as a proxy for relative rodent abundance, and Simpson’s diversity index [32].

Dried blood samples were used for the detection of *T. gondii* antibodies using a modified agglutination test (MAT) technique [9] adapted for dried blood samples, with a cut-off titre at 1:16 [20]. Two 5 mm diameter dried blood spots were punched out of each blotting paper circle and placed into the well of a flat bottomed microtitre plate. The blood was eluted out in 80 μL of phosphate buffered saline, pH 7.2 (bioMérieux). Plates were covered and left to elute overnight at RT and at 400 rpm agitation. Ten microlitres of each eluted sample were used. Samples were screened at four serial dilutions (1:16, 1:32, 1:320 and 1:640). For serological control, fresh blood from *T. gondii* antibody-seronegative Swiss mice (not infected by *T. gondii*) and *T. gondii* antibody-seropositive Swiss mice (experimentally infected with control of the presence of cysts in the brain) (*Mus musculus*, Janvier Labs, Saint-Berthevin, France) was spotted onto a 5 mm diameter circle on Whatman No. 3 paper and allowed to dry at RT for 24 h, before storage at RT in sealed bags. Antibody titres were determined by the last dilution where the agglutination pattern could be read in comparison with the negative and positive controls. *Toxoplasma* seroprevalence was finally calculated as the percentage of seropositive rodents in each sampling locality.

### Statistical analyses

We carried out a generalised linear model (GLM) assuming a binomial distribution to test whether individual rodent seropositivity was related to geography (described by latitude and longitude coordinates of the sampled individual), rodent community diversity (Simpson index) and relative abundance (estimated using trap success) at the locality of sampling, as well as rodent species, sex and body mass (this variable was centred-reduced to avoid the confounding effect of specific differences). The interactions between rodent species and geographical coordinates were also considered in the model. Using the Akaike information criterion with correction for samples of finite size (AICc), we carried out model selection from the full starting model. We then chose the most parsimonious model among those selected within two AIC units of the best model obtained. *P* values were obtained by stepwise model simplification using likelihood-ratio tests and were considered significant when *p* < 0.05. The final model was validated by the graphical checking of normality, independence and variance homogeneity of the residuals. All analyses were performed with R Software using the lme4 v1.1-8 [4] and MuMIn v1.15.1 [3] packages.

## Results

A total of 1604 rodents were sampled (mean trap success: 0.30 ± 0.11): 689 house mice (*Mus musculus domesticus*), 513 multimammate rats (376 *Mastomys erythroleucus* and 137 *M. natalensis*), 334 black rats (*Rattus rattus*), 39 Nile rats (*Arvicanthis niloticus*), 25 Dalton’s mice (*Praomys daltoni*), three brown rats (*Rattus norvegicus*) and one fat mouse (*Steatomys* sp.) (Table 1). Rodent diversity was relatively low within localities (mean Simpson’s diversity index: 0.78 ± 0.21) (Table 1).

A total of 1205 rodents (*Mastomys* spp., *R. rattus*, *M. m. domesticus*, *A. niloticus* and *P. daltoni*) were screened for *Toxoplasma* antibodies. Among them, we found 44 seropositive rodents (Table 1). MAT titres were: 1:16 in nine, 1:32 in 28, and above 1:320 in seven. The 44 seropositive rodents belonged to four different species: the native *M. erythroleucus* (*n* = 13; 4% of the screened individuals) and *M. natalensis* (*n* = 2; 2%), and the invasive *M. m. domesticus* (*n* = 25; 5%), and *R. rattus* (*n* = 4; 2%). No seropositive individuals were found among *A. niloticus* and *P. daltoni*, represented by very low sample sizes (*n* = 23 and *n* = 18, respectively). Within localities, *Toxoplasma* seroprevalence (mean: 0.04 ± 0.06) ranged from zero (20 localities) to 24% (code THC) (Fig. 1; Table 1).

Statistical analysis was restricted to data for the four species with sufficient sample sizes (i.e., *M. m. domesticus*, *R. rattus* and *Mastomys* species). Rodent seropositivity was significantly related to latitude (Likelihood Ratio Test LRT_1,1164_ = 7.91 *p* = 0.0049), with higher seroprevalence in northern localities (Fig. 1), but not with longitude (LRT_1,1164_ = 0.57, *p* = 0.4520), Simpson diversity (LRT_1,1164_ = 0.01, *p* = 0.9346), trap success (LRT_1,1164_ = 0.22, *p* = 0.6423), rodent species (LRT_3,1164_ = 0.96, *p* = 0.8103), sex (LRT_1,1164_ = 0.64, *p* = 0.4254) or body mass (LRT_1,1164_ = 0.64, *p* = 0.4254).

## Discussion

In Senegal, commensal rodent communities are dominated by native *Mastomys* spp., as well as by the invasive *M. m. domesticus* from the centre to the north of the country, or by *R. rattus* in the southern part [7] (Fig. 1). The distribution of the various species reflects the ongoing eastwards spread of invasive rodents from coastal localities where they were introduced during the colonial era [7, 15, 18].

Mean *Toxoplasma* seroprevalence was relatively low (4%) in rodents from Senegal. This value closely matches that found in rodents elsewhere [2, 13, 19, 20, 22]. Low seroprevalence in rodents led to an initial question on the sensitivity of the serological test used to detect *T. gondii* infection. The MAT technique is commonly used in diverse species of animals. It was shown to be a reliable indicator of infection in mice of several laboratory strains [11, 26]. Seronegative values were nevertheless reported in *Mus musculus* offspring infected by vertical transmission from chronically infected dams, although the infection was confirmed by PCR [5, 27]. Diverse virulence of a given *T. gondii* strain has been observed in laboratory and wild mouse strains, indicating different innate immunological reactions linked to polymorphic variations in the IRG system [16, 21]. Also, differences in antibody response against *T. gondii* infection among wild rodent species cannot be excluded. All these elements point out the benefit of combining different techniques of *Toxoplasma* detection in future studies, in order to evaluate their respective value for the screening of rodent communities in the wild.

Low *Toxoplasma* seroprevalence is expected under Sahelian climatic conditions (low hygrometry, high soil and air temperatures and ultraviolet irradiation levels), which are poorly suitable for oocysts survival and sporulation [10] and may decrease the probability of environmental contamination [33]. On the basis of this assumption, mean seroprevalence was found to be similarly low in this study and in the only other rodent survey performed in the Sahel (e.g., in Niamey, Niger: <2% *Toxoplasma* seroprevalence in *M. m. domesticus*, *R. rattus* and *M. natalensis*) [20]. Nevertheless, climatic conditions cannot explain the latitudinal gradient found in Senegal. Indeed, in this country the main climatic factor related to latitude is aridity, which would be unfavourable to *Toxoplasma* transmission but increases from south to north [29] like the number of positive rodents. This unexpected latitudinal pattern suggests the effect of environmental factors other than climate on *Toxoplasma* seroprevalence in rodents. For instance, the proximity of irrigated surfaces may play a role, as indicated by higher *Toxoplasma* seroprevalence found in rodents that were sampled within irrigated gardens in Niamey [20]. Seroprevalence values in rodents may also reflect differences in the distribution of other host species such as cats, dogs and livestock, which were found to be largely infected by *Toxoplasma* in Senegal [8, 12, 28]. For instance, the higher number of positive rodents in the North could relate to the large livestock populations of the Ferlo region, which is the heart of pastoral herding in Senegal [6]. Large populations of domestic cats have been observed in villages in the Senegal River’s middle valley (Duplantier, unpublished data), but there are unfortunately no data on the distribution of domestic and wild felids across the country that could help to explain the differences in *Toxoplasma* seroprevalence found in rodents among the different localities [8].

Variations in *T. gondii* infection were not related to variations in rodent communities among localities (Fig. 1). We would have expected to detect higher seroprevalence values in some species than in others because of differences in body size and home range size (e.g., *R. rattus* larger than *M. m. domesticus*), or in some individuals because of sex or body mass (e.g., [1, 30]). However, low seroprevalence levels lead to low statistical power to detect the effect of such factors in complex models. Because of their shorter life expectancy and smaller home-range, rodents may be less likely to be infected than larger mammals [1], which raises the question of their role as sentinels for *T. gondii* infection. The specific ecological characteristics of the rodents sampled in this study could also explain the low seroprevalence. Indeed, *R. rattus* (that often build nests in the roofs of houses), or *M. m. musculus* and *Mastomys* species (that use crevices or cracks as burrows) are less at risk of being infected by *T. gondii* than fossorial mammals, because they are less in contact with potentially contaminated soil [1].

To our knowledge, this study is the first to focus on *Toxoplasma* seroprevalence in rodents at the scale of a Sahelian country. To obtain a clearer view of *T. gondii* epidemiology, similar studies in rural areas on other epidemiological agents such as cats, sheep and goats, are necessary. They will enable us to better understand the risk factors related to *T. gondii* in villages and towns of Senegal, which seem to be variable geographically.

## Conflict of interest

The authors declare that they have no conflict of interest.
